# Evaluation and comparison of patient satisfaction with VertexThermosens and conventional acrylic complete dentures

**DOI:** 10.1002/cre2.829

**Published:** 2024-01-18

**Authors:** Sebahate H. Alidema, Rajmonda Halili

**Affiliations:** ^1^ Department of Prosthodontics Alma Mater Europaea Campus College Rezonanca Prishtina Kosovo; ^2^ Department of Prosthodontics AAB College Prishtina Kosovo

**Keywords:** complete dentures, edentulous, oral health

## Abstract

**Objectives:**

Patient perception and satisfaction with dentures are important indicators in prosthodontic treatment. This study aimed to compare patients' satisfaction with VertexThermosens dentures versus conventional acrylic dentures during a 12‐month study period.

**Material and Methods:**

This randomized study involved 60 participants aged between 45 and 80 with representation of both sexes. The patients were divided into two groups: The first group (experimental group), which had complete dentures from VertexThermosens base material included 30 participants. The second group (control group), which had complete dentures from convectional rigid acrylic base material, included 30 participants. Patient satisfaction with the dentures was assessed in each of the two Vertex/Acrylic groups through a specially created questionnaire of five questions with a five‐point Likert scale of possible answers (0 = *never*; 1 = *rarely*; 2 = *occasionally*; 3 = o*ften*; and 4 = *very often*).

**Results:**

After 12 months, intergroup comparison with Mann–Whitney *U* Test related to Q2 (*p* = 0.193), Q3 (*p* = 0.960), Q4 (*p* = 0.317), Q5 (*p* = 1.000) patient satisfactions indicated that there was no significant difference between the two groups (Vertex/Acrylic), except  Q1 (*p* = 0.010) question, Vertex group was more satisfied. The results regarding the patients of both groups (Vertex/Acrylic) showed that after 12 months of wearing the denture, the satisfaction level regarding to all questions increased, except Q5‐question for Vertex group, where the difference was not significant due to the already achieved maximum satisfaction at zero time.

**Conclusion:**

Based on the findings of this clinical study, it can be concluded that higher satisfaction is seen in patients rehabilitated with VertexThermosens dentures than with conventional rigid acrylic dentures.

## 1 INTRODUCTION

Although the introduction of dental implants made it possible to replace conventional dentures with implant‐retained dentures, according to studies, prosthodontic treatment with complete dentures (CD) is still one of the most suitable methods for rehabilitation of edentulous patients considering their health and socioeconomic status (Oweis et al., 2022; Soboleva & Rogovska, 2022). Treatment with CD exerts an impact on the quality of oral health, which also affects the quality of life (QoL) (Limpuangthip et al., 2019).

The patient observation and satisfaction are as important indicators as clinical ones and can impact the success of the treatment plan of edentulous patients (Albaker, 2013). Considering the adaptive ability of patients with dentures is different, the comfort of patients with dentures depends to a large extent on the psychological factor. Some of the complaints coming from patients, even when the dentures meet all the clinical and technical conditions, is the inability to eat well and the poor stability of the dentures.

Numerous factors impact patient satisfaction with CD, such as age, gender, level of education, patient expectation of dentures, psychological characteristics of patients, patient–dentist communication issues, quality of dentures (retention, stability, fit, vertical dimension, occlusion, different denture base material, denture manufacturing technique and protocol, arrangement of teeth, and esthetics), occlusal factors, class of alveolar ridge, saliva (viscous or serous), the size of the tongue and xerostomia (Alves et al., 2018; Budală et al., 2021; Celebic et al., 2003; Darwish, 2021; Hatim & Mohi Al–Deen, 2014; Limpuangthip et al., 2019; Marchini, 2014; Musavi et al., 2018; Ohara et al., 2022; Oweis et al., 2022; Possebon et al., 2020; Soboleva & Rogovska, 2022). Several questionnaires have been created to assess the correlation between the above factors and oral health with QoL. One of the most widely used questionnaire instrument to measure the impact of oral health on the QoL is Oral Health Impact Profile‐49 (OHIP‐49). To shorten the time for filling out the questionnaire, it was compiled a shorter version of OHIP, the OHIP‐14, which is considered quite suitable and is available in several languages in Europe and worldwide (Bimbashi et al., 2012; Kołciuk & Godlewski, 2015; Montero‐Martín et al., 2009; Rener‐Sitar et al., 2008). Moreover, for edentulous patients another specific version of the OHIP was prepared, such as OHIP Edentulous (OHIP‐EDENT) (Allen & Locker, 2002). In addition, the use of OHIP‐7T and OHIP‐5 with a lesser number of questions made it possible to assess oral health related to QoL (OHRQoL) in almost any dimension by simplifying the procedure, therefore making it less annoying for the patient, and a comprehensive OHRQoL assessment with a good informative result has been achieved (Naik et al., 2016; Teng et al., 2020). The satisfaction regarding CD, that the patients are usually asked to assess include difficulties in chewing food, speech problems, ability to taste food or feeling a bad taste, comfortability, and esthetically.

The satisfaction level of CD patients is highly dependent on the dentist‐patient relationship. The advice given to the patient should be easy to understand depending on the patient's age and intellectual level. Factors such as the patient's personality and the dentist–patient interaction can greatly affect the success of prosthetic treatment with CD. However, the nature of denture acceptance is highly subjective. In many cases, the dentist may have difficulty persuading the patient to wear dentures that are well‐fitted due to psychological factors (Pasad et al., 2021). Anxiety is a factor that greatly affects CD satisfaction of edentulous patients (Shrivastava et al., 2012).

According to studies, to improve the quality of dentures modifications have been made to denture base materials, including conventional acrylic resins, high‐impact resins, glass fiber‐reinforced resins, and metal‐reinforced resins (Lamfon & Hamouda, 2019). These modifications have been made to improve the QoL of patient's denture wearers as much as possible. Rigid acrylic dentures do not always meet functional and esthetic requirements. As most patients wearing CD are elderly and in poor health, they complain of injuries caused by the rigid base of the dentures, so perfect materials are always required. According to the studies thermoplastic materials are a better choice for dentures, taking into account their flexible properties and resistance to breakage (Hazari et al., 2015; Im & Am, 2018). The disadvantage of thermoplastic dentures is the fact that over time their surface becomes rougher and loses its luster, which makes staining and discoloration easier. They are also a suitable place for buildup of plaque and growing bacteria if the wearer doesn't maintain proper oral hygiene.

Although various studies have been done on the esthetic, retention, masticatory performance, and comfort of dentures, objective studies that evaluate and compare patient satisfaction with conventional acrylic CD dentures and thermoplastic VertexThermosens CD are lacking. Therefore, the aim of this study was to assess and compare the impact of these two types of denture materials on the QoL of patients in the 12 months of denture use.

## 2 METHODS

This randomized study was carried out at the Department of Prosthetics “Alma Mater Europaea Campus College” “REZONANCA” in Prishtina, Kosovo with prior approval from the Ethical Committee (AD‐3063/21,18.06.2021).

A comparative clinical trial involved 60 participants aged between 45 and 80 with representation of both sexes. The patients were randomly divided in two groups: the first group (experimental group)—30 participants had CDs from VertexThermosens base material; the second group (control group)—30 participants had CDs from Convectional Rigid Acrylic base material.

The exclusion criteria were the following: radiation, immunosuppressive therapy, bone metabolic diseases, allergy in denture base material.

Patient satisfactions with the dentures were assessed in each of the two Vertex/Acrylic groups through the OHIP5 questionnaire with a five‐point Likert scale of possible answers (0 = *never*; 1 = *rarely*; 2 = *occasionally*; 3 = *often*; 4 = *very often*). The questions covered the following aspects: (a) Q1—chewing difficulties (Have you had difficulty chewing any foods because of problems with your teeth, mouth, dentures or jaw?); (b) Q2—pain/discomfort (Have you had painful aching in your mouth?); (c) Q3—awareness/concern about mouth problems (Have you felt uncomfortable about the appearance of your teeth, mouth dentures or jaws?); (d) Q4—feeling of bad taste (Have you felt that there has been less flavor in your food because of problems with your teeth, mouth, dentures or jaws?); and (e) Q5—difficulties in performing daily activities due to a problem with the mouth (Have you had difficulty doing your usual jobs because of problems with your teeth, mouth, dentures, or jaws?). A lower total score indicated greater patient satisfaction. The questionnaire was applied two times, at the first week of denture insertion (zero) and after 12 months of denture insertion.

### 2.1 Statistical analysis

The data obtained with the research were processed in SPSS software package, version 22.0 for Windows, and presented in tables and figures. The qualitative series were processed by determining the coefficient of relations, proportions, and rates, and were shown as absolute and relative numbers. Quantitative series were analyzed with measures of central tendency (mean, median, minimum, and maximum values, interactive ranks), as well as by dispersion measures (standard deviation). Two independent numerical variables with nonnormal distribution of frequencies were compared with the Mann–Whitney *U* test. The analysis of two and more dependent variables with nonnormal distribution was made with Wilcoxon signed rank test.

A two‐sided analysis with a significance level of *p* < 0.05 was used to determine the statistical significance.

## 3 RESULTS

The analysis of general characteristics referred to the distribution of patients from both groups according to gender and age.

**Figure 1:**
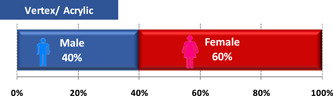
Analysis of Vertex/Acrylic groups according to gender.

**Gender**: In each of the two groups (Vertex/Acrylic), an analysis was made of the distribution of patients according to gender (Figure 1). The presence of men and women in groups with different types of CD (Vertex/Acrylic) was equal for 12 (40%) versus 18 (60%) respectively with a gender ratio of 0.66:1.

The percentage differences of the presence of men compared to women in both groups were insignificant (difference test: 6%, 95% CI: [−12.83 to 24.63]); *χ*² = 0.416; *df* = 1; *p* = 0.519).

**Age**: The average age of the patients in the Vertex group was 61.40 ± 8.96 with a min/max of 45/70 years, and in the acrylic group it was 65.20 ± 9.04 with a min/max of 45/79 years. The analysis indicated that 50% of patients in the Vertex or Acrylic group were younger than 61 years for median (interquartile range [IQR]) = 61 (57–67) versus 65 years for median (IQR) = 65 (59–72) (Table 1).

**Table 1:** Analysis of Vertex/Acrylic groups according to age.

Parameters	Age (years)				*p* *
	*N*	Mean ± SD	Min/Max	Median (interquartile range)	
Groups (*N* = 60)					
Vertex	30	61.40 ± 8.96	45/70	61 (57–67)	*T*‐test _(58)_ = 1.591; *p* = 0.1169
Acrylic	30	65.20 ± 9.04	45/79	65 (59–72)	
Vertex (*N* = 30)					
Male	12	63.17 ± 8.45	46/79	62 (59–69.5)	*T*‐test _(28)_ = −0.878; *p* = 0.3874
Female	18	60.22 ± 9.33	49/79	59 (53–65)	
Acrylic (*N* = 30)					
Male	12	68.17 ± 7.81	56/79	69.5 (62–74.5)	*T*‐test _(28)_ = 1.552; *p* = 0.1317
Female	18	63.05 ± 9.43	45/79	64 (57–67)	

* Significant for *p* < 0.05.

To see the internal consistency of the block of five questions about patient satisfaction, a reliability analysis was made of the answers received at both times (zero and 12 months) by calculating the coefficient Cronbach's *α* (Table 2). The results showed that the coefficient Cronbach's *α* is 0.773 versus 0.707 respectively, indicating satisfactory reliability of received answers from patients (Table 2).

**Table 2:** Internal consistency of patient satisfaction in two time points.

Time of testing	Cronbach's *α*	Cronbach's *α* based on standardized items	Total questions^a^
Zero (first week)	0.773	0.767	5
12 months	0.707	0.730	5

^a^ Questioner for patients' satisfaction.

**Figure 2:**
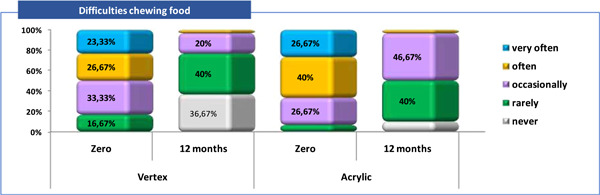
Patient satisfaction related to “Difficulties chewing food” (Q1).

**Figure 3:**
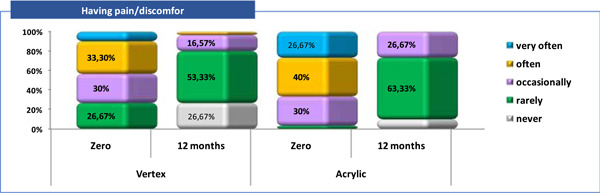
Patient satisfaction related “Pain/discomfort” (Q2).

**Figure 4:**
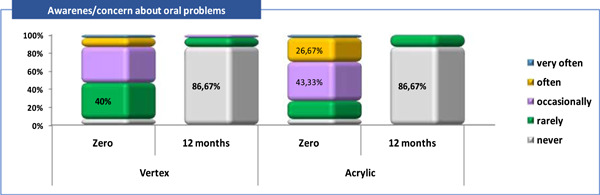
Patient satisfaction related to “Awareness/concern about oral problems” (Q3).

**Figure 5:**
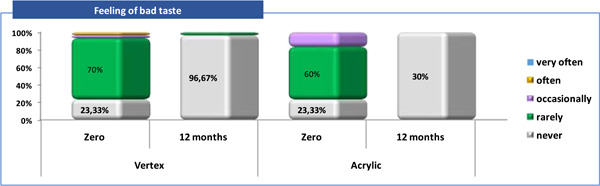
Patient satisfactions related to “Feeling of bad taste” (Q4).

**Figure 6:**
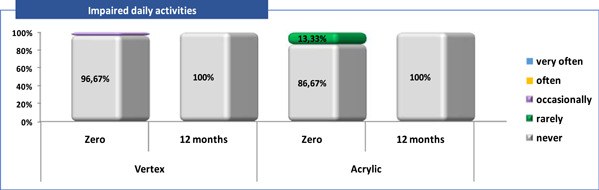
Patient satisfaction related to “Impaired daily activities” (Q5).

A descriptive analysis was made according to the category of answers received to each of the five questions about patient satisfactions and it was observed that (Tables 3, 4 and Figures 2, 3, 4, 5, 6).

**Table 3:** OHIP‐5 values of the respective dimensions in relation to time: First week and after 12 months of Vertex and Acrylic denture use.

Questions^a^		Likert scale for satisfaction^b^				
		Never	Rarely	Occasionally	Often	Very often
1. Difficulties chewing food?—*N* (%)						
Zero	Vertex	0 (0%)	5 (16.67%)	10 (33.33%)	8 (26.67%)	7 (23.33%)
	Acrylic	0 (0%)	2 (6.67%)	8 (26.67%)	12 (40%)	8 (26.67%)
12 months	Vertex	11 (36.67%)	12 (40%)	6 (20%)	1 (3.33%)	0 (0%)
	Acrylic	3 (10%)	12 (40%)	14 (46.67%)	1 (3.33%)	0 (0%)
2. Having pain/discomfort?—*N* (%)						
Zero	Vertex	0 (0%)	8 (26.67%)	9 (30%)	10 (33.3%)	3 (10%)
	Acrylic	0 (0%)	1 (3.33%)	9 (30%)	12 (40%)	8 (26.67%)
12 months	Vertex	8 (26.67%)	16 (53.33%)	5 (16.57%)	1 (3.33%)	0 (0%)
	Acrylic	3 (10%)	19 (63.33%)	8 (26.67%)	0 (0%)	0 (0%)
3. Awareness/concern about oral problems?—*N* (%)						
Zero	Vertex	2 (6.67%)	12 (40%)	12 (40%)	3 (10%)	1 (3.33%)
	Acrylic	2 (6.67%)	6 (20%)	13 (43.33%)	8 (26.67%)	1 (3.33%)
12 months	Vertex	26 (86.67%)	3 (10%)	1 (3.33%)	0 (0%)	0 (0%)
	Acrylic	26 (86.67%)	4 (13.33%)	0 (0%)	0 (0%)	0 (0%)
4. Feeling of bad taste?—*N* (%)						
Zero	Vertex	7 (23.33%)	21 (70%)	1 (3.33%)	1 (3.33%)	0 (0%)
	Acrylic	7 (23.33%)	18 (60%)	5 (16.67%)	0 (0%)	0 (0%)
12 months	Vertex	29 (96.67%)	1 (3.33%)	0 (0%)	0 (0%)	0 (0%)
	Acrylic	30 (100%)	0 (0%)	0 (0%)	0 (0%)	0 (0%)
5. Impaired daily activities?—*N* (%)						
Zero	Vertex	29 (96.67%)	0 (0%)	1 (3.33%)	0 (0%)	0 (0%)
	Acrylic	26 (86.67%)	4 (13.33%)	0 (0%)	0 (0%)	0 (0%)
12 months	Vertex	30 (100%)	0 (0%)	0 (0%)	0 (0%)	0 (0%)
	Acrylic	30 (100%)	0 (0%)	0 (0%)	0 (0%)	0 (0%)

^a^ Questioner for patients' satisfaction.

^b^ Likert scale—(0 = *never*; 1 = *rarely*; 2 = *occasionally*; 3 = *often*; 4 = *very often*).

**Table 4:** Comparison of the OHIP‐5 values of the respective dimensions in relation to time: First week and after 12 months of Vertex and Acrylic denture use.

Questions	Likert scale for patient satisfactions^a^					
	Zero^b^			12 months		*p* c
	Number (*N*)	Mean ± SD	50th (Median)	Mean ± SD	50th (Median)	
1. Difficulties chewing food?—*N* (%)						
Vertex	30	2.57 ± 1.04	2.5	0.90 ± 0.84	1.0	*Z* = −4.782; *p* = 0.0001^d^
Acrylic	30	2.87 ± 0.89	3.0	1.43 ± 0.73	1.5	*Z* = −4.748; *p* = 0.0001^d^
*p* e		*Z* = −1.157; *p* = 0.247		*Z* = −2.575; *p* = 0.010		**‐**
2. Having pain/discomfort?—*N* (%)						
Vertex	30	2.27 ± 0.98	2.0	0.97 ± 0.76	1.0	*Z* = −4.584; *p* = 0.0001^d^
Acrylic	30	2.90 ± 0.84	3	1.67 ± 0.59	1.0	*Z* = −4.788; *p* = 0.0001^d^
*p* e		*Z* = −2.451; *p* = 0.014		*Z* = −1.301; *p* = 0.193		**‐**
3. Awareness/concern about oral problems?—*N* (%)						
Vertex	30	1.63 ± 0.89	2	0.17 ± 0.46	0	*Z* = −4.736; *p* = 0.0001^d^
Acrylic	30	2.00 ± 0.95	2	0.13 ± 0.34	0	*Z* = −4.710; *p* = 0.0001^d^
*p* e		*Z* = −1712; *p* = 0.087		*Z* = −0.050; *p* = 0.960		**‐**
4. Feeling of bad taste?—*N* (%)						
Vertex	30	0.87 ± 0.63	1	0.33 ± 0.18	0	*Z* = −4.630; *p* = 0.0001^d^
Acrylic	30	0.93 ± 0.64	1	0.00 ± 0.00	0	*Z* = −4.460; *p* = 0.0001^d^
*p* e		*Z* = −0.561; *p* = 0.575		*Z* = −1.000; *p* = 0.317		**‐**
5. Impaired daily activities?—*N* (%)						
Vertex	30	0.07 ± 0.36	0	0.00 ± 0.00	0	*Z* = −1.000; *p* = 0.317^d^
Acrylic	30	0.13 ± 0.34	0	0.00 ± 0.00	0	*Z* = −2.000; *p* = 0.046^d^
*p* e		*Z* = −1.327; *p* = 0.185		*Z* = −0.000; *p* = 1.000		**‐**

^a^ Likert scale—(0 = *never*; 1 = *rarely*; 2 = *occasionally*; 3 = *often*; 4 = *very often*).

^b^ Zero: First week after intervention.

^c^ Wilcoxon signed ranks test.

^d^ Significant for *p* < 0.05.

^e^ *Z* = Mann–Whitney *U* test.

Q1—difficulties chewing food: in zero time, the majority of patients from Vertex/Acrylic groups answered that they have chewing difficulties “often” for 8 (26.7%) versus 12 (40%) respectively, and “very often” for 7 (23.3%) versus 8 (26.7%) respectively. None of the patients in the both groups answered that they “never” had a problem with chewing. After 12 months, this problem had “never” versus “rarely” 11 (36.7%) versus 12 (40%) of the patients with Vertex and 3 (10%) versus 12 (40%) of the one with Acrylic respectively (Tables 3, 4 and Figure 2).

Q2—pain/discomfort: at zero time, the majority of patients from Vertex/Acrylic group answered that they had pain/discomfort “often” in 10 (33.3%) versus 12 (40%) respectively and “very often” in 3 (10%) versus 8 (26.7%) respectively (Tables 3, 4 and Figure 3). None of the patients in both groups answered that “never” had pain/discomfort. After 12 months, “never” or “rarely” had this problem 8 (26.7%) versus 16 (53.3%) of the patients with Vertex and 3 (10%) versus 19 (63.3%) of the one with Acrylic.

Q3—awareness/concern about the oral problems: at zero time, in each of the groups (Vertex vs. Acrylic), “occasionally” concerned about the oral problems were 12 (40%) versus 13 (43,3%) of the patients, and “often” worried were 3 (10%) versus 8 (26.7%) of them respectively. Only one (3.3%) patient from both groups had this problem “very often” at zero time. After 12 months, “never” or “rarely” had this problem, an equal proportion of patients from each of the groups, or 26 (86,7%) versus 3 (10%) respectively (Tables 3, 4 and Figure 4).

Q4—feeling of bad taste: in zero time, the majority of patients from both groups (Vertex/Acrylic) answered—7 (23,3%) that they “never” had feeling of bad taste. This sensation at zero time was “rare” in 21 (70%) of the patients with Vertex and 18 (60%) of those with Acrylic. After 12 months, without a feeling of bad taste were 29 (96.7%) of the patients with Vertex and all 30 (100%) patients with acrylic (Tables 3, 4 and Figure 5).

Q5—impaired daily activities: almost all patients with Vertex/Acrylic, 29 (96.7%) versus 26 (86.7%) respectively, answered that they “never” had their daily activities disrupted due to the dentures. After 12 months, all 30 (100%) patients from both groups “never” had this feeling.

The results regarding the patients of both groups (Vertex/Acrylic) showed that after 12 months of wearing the denture, the satisfaction level regarding to all questions increased, except Q5—question for Vertex group, where the difference was not significant due to the already achieved maximum satisfaction at zero time (Tables 3, 4 and Figure 6).

Additionally, intergroup comparison with Mann–Whitney *U* test related to Q1 (*Z* = −1.157; *p* = 0.247), Q3 (*Z* = −1.712; *p* = 0.087), Q4 (*Z* = −0.561; *p* = 0.575), Q5 (*Z* = −1,327; *p* = 0.185) patient satisfactions indicated that there was no significant difference between the two groups (Vertex/Acrylic) in first week of wearing dentures, except Q2 question, Vertex group was more satisfied (*Z* = −2.451; *p* = 0.014) (Tables 3, 4 and Figures 3, 4, 5, 6). Meanwhile, the comparison between the two groups (Vertex/Acrylic) related to Q2 (*Z* = −1.301; *p* = 0.193), Q3 (*Z* = −0.050; *p* = 0.960), Q4 (*Z* = −1.000; *p* = 0.317), Q5 (*Z* = −0.000; *p* = 1.000) patient satisfaction revealed a nonstatistically significant difference in mean satisfaction score after 12 months. Nevertheless, related to Q1 patient satisfactions a statistically significant difference in mean satisfaction score was found at Vertex group (*Z* = −2.575; *p* = 0.010) (Tables 3, 4 and Figures 3, 4, 5, 6).

## 4 DISCUSSION

Determining the factors that influence the satisfaction of patient denture wearers is of great importance, considering that it also affects overall health and QoL of the patient. Objective professional viewpoints do not always correspond to patient satisfaction. Therefore, this study focused on patient evaluation dentures from the patient's viewpoints by answering five questions, the responses to which were rated on 0–4 scale, from never to very often (0—*never*, 1—*rarely*, 2—*occasionally*, 3—*often*, 4—*very often*).

Improvement questionnaire score in both groups after 12 months of denture wearing reflects improved patient satisfaction. Similar findings have been reported by other authors, although by using other questionnaire instruments (Čelebić & Knezović‐Zlatarić, 2003; Km et al., 2021; Musavi et al., 2018; Mushtaha et al., 2020; Nazeer et al., 2016). In addition, improvement was observed also in clinical objective status. In the first month of dentures, patients had lesions in the form of minor injuries which were healed after denture corrections with systematic appointments. By comparing the two groups, statistically there was an insignificant difference in patient satisfaction after 1 week of denture insertion with regard to questions related to the “difficulties chewing food” (Q1), “awareness/concern about the oral problems” (Q3), “feeling bad taste” (Q4), “impaired daily activities” (Q5). Whereas, in regard to the question related to “pain/discomfort” (Q2) the VertexThermosens dentures group was significantly more satisfied than conventional acrylic group. This may be due to thinner, lighter layer and small flexibility of thermoplastic VertexThermosens denture base material. Interestingly, after 12 months of denture insertion, conventional acrylic group showed improvement of satisfaction in the question related to “pain/discomfort” (Q2), although VertexThermosens group had higher percentage of responses in 0 and 1 scale than the acrylic group, which had more responses in 1 and 2 scale. The ability of denture base materials to withstand force of mastication depends on flexural strength, which is higher in VertexThermosens denture base materials, and it simulates the stress types that are applied on denture base during mastication, which cause less injury (Mohsin et al., 2017). The improvement of acrylic denture wearers related to “pain/discomfort” may have occurred because the base of the acrylic dentures is more rigid and has led to more noticeable injury of the oral mucosa, while with further adaptation of the oral mucosa, the injury has been reduced. Moreover, the higher level of patient satisfaction can also be attributed to regular invitations for visits, which makes the patient feel better treated. Similar results were reported by other studies (Ellis et al., 2007; John et al., 2004; Kołciuk & Godlewski, 2015; Mushtaha et al., 2020).

In the present study, after 12 months, intergroup comparison with Mann–Whitney *U* test related to Q2, Q3, Q4, and Q5 satisfaction indicated that there was no significant difference between the groups. Related to the “difficulties of chewing food” question, VertexThermosens dentures group was significantly more satisfied than the conventional acrylic group. Nevertheless, it needs to be highlighted that both groups had significant improvement in chewing food with passing of time. In the present study, patient self‐evaluation satisfaction is in agreement with clinical evaluation related to “chewing ability” as reported in other studies (Al‐Jammali & Al Nakkash, 2013; Fayad et al., 2018; Hazari et al., 2015; Kar et al., 2019). Fayad et al. in their study reported a significant increase of the bite force after 6 months of denture wearing in the thermoplastic dentures group compared to conventional acrylic dentures group. However, at the time of denture insertion, these two groups did not show any significant difference in bite force. In addition, bite force was increased considerably after 6 months of denture use in both groups (Fayad et al., 2018), which corroborate the findings of the present study and are in agreement with the results of other studies as well (Borie et al., 2014; Km et al., 2021).

According to a study (Hazari et al., 2015), a statistically significant number of patients found flexible dentures to be more satisfying and comfortable than conventional dentures. These findings were in accordance with other studies (Akinyamoju et al., 2017; Dhiman & Chowdhury, 2009; Goiato et al., 2010). In present study, even though there were no significant differences between the groups, it was validated that from the start of the denture insertion, the patient's satisfaction scored higher in the evaluation scale for the VertexThermosens group. The use of VertexThermosens CD is an effective treatment from the viewpoint of patient preference. Most patients seem to have more comfortable dentures and better chewing ability with this denture base material.

The limitations of the current study are that the observations were made over a short period of time (12 months) and patients who had CD insertions for the first time were not distinguished from patients who already had them. Further studies with a longer observation period and a larger sample size can be conducted to reach a more definitive conclusion whether the VertexThermosens or conventional rigid acrylic dentures are a better choice.

## 5 CONCLUSION

No significant statistical difference in patient satisfactions was observed after rehabilitation with thermoplastic VertexThermosens and conventional rigid acrylic dentures at the end of 12 months observation period, with the exception of the “difficulties chewing food” question whereby VertexThermosens dentures group was significantly more satisfied than conventional Acrylic group. There was a significant improvement in patients' satisfaction with dentures in both groups within the period 0–12 months.

## AUTHOR CONTRIBUTIONS

All authors made substantial contributions to the study concept, the analysis, and interpretation of the data. Moreover, they drafted the manuscript, revised it critically and approved the final version of the manuscript to be published.

## CONFLICT OF INTEREST STATEMENT

The authors declare no conflict of interest.

## ETHICS STATEMENT

The study was conducted in accordance with the Declaration of Helsinki and approved by the Ethical Committee of Alma Mater Europaea Campus College Rezonanca, Pristina, Kosovo (AD‐3063/21,18.06.2021).

## Data Availability

The data that support the findings of this study are available upon request from the corresponding author.
